# Psychometric Properties of the Generalized Anxiety Disorder Scale-7 Item (GAD-7) in a Large Sample of Chinese Adolescents

**DOI:** 10.3390/healthcare9121709

**Published:** 2021-12-09

**Authors:** Jiangang Sun, Kaixin Liang, Xinli Chi, Sitong Chen

**Affiliations:** 1 College of Physical Education, West Anhui University, Lu’An 237012, China; sjgsport@163.com; 2 School of Psychology, Shenzhen University, Shenzhen 518060, China; liangkaixin2020@email.szu.edu.cn (K.L.); xinlichi@126.com (X.C.); 3 Institute for Health and Sport, Victoria University, Melbourne 8001, Australia

**Keywords:** anxiety, psychometrics, measurement, GAD-7, generalized anxiety disorder scale, adolescents, students

## Abstract

Anxiety symptoms are common among adolescents. A well-validated and easy-to-use tool is indispensable to measure and detect anxiety for timely interventions. The Generalized Anxiety Disorder Scale-7 item (GAD-7) is a self-report scale used to measure the severity of anxiety and has been validated in adult populations, but psychometric properties of the GAD-7 remained rarely tested in adolescents. The study aimed to investigate the reliability and validity of the GAD-7 in Chinese adolescents. Sex- and age-specific analyses were conducted in a large sample of adolescents (*n* = 67,281, aged 10–17 years). Our results showed that the GAD-7 scores were higher in female and older adolescents. The GAD-7 presented good internal consistency and a unidimensional structure across sex- and age-specific groups. The GAD-7 scores were significantly correlated with the scores of the Patient Health Questionnaire-9 item (PHQ-9, a self-reported scale to measure depression symptoms) in all subgroups, indicating acceptable criterion validity. In conclusion, the GAD-7 is a scale with good psychometrics and can serve as a tool for anxiety screening in Chinese adolescents at the populational level.

## 1. Introduction

Anxiety is one of the most concerning mental health problems across the world affecting various populations [1], including adolescents. According to the global data, 6.5% of children and adolescents are suffering from anxiety disorders [2]. Moreover, under the context of the COVID-19 pandemic, the pooled estimate obtained in the first year of the COVID-19 pandemic suggests that one in five youths was experiencing clinically elevated anxiety symptoms [3]. Some studies even reported that the prevalence of anxiety symptoms reached more than 30% among adolescents during the COVID-19 pandemic [4,5]. Furthermore, anxiety disorders in adolescents can have long-term consequences on the quality of life and are often comorbid with other problems, such as depression, sleep disturbances, and attention-deficit/hyperactivity disorder [6,7,8,9]. Despite the fairly high prevalence and the psychosocial impairment that comes with anxiety disorders, the public generally has a relatively low understanding of symptoms, and anxiety disorders or symptoms often remain undetected and untreated [10]. Therefore, it is crucial to have a suitable screening tool to help measure and identify anxiety among adolescents for subsequent treatment as early as possible. A suitable screening instrument should be well-validated, brief, easy to administer and score, able to be interpreted without extensive and professional training, feasible in practice, and free to use if possible.

The Generalized Anxiety Disorder-7 item (GAD-7) is a brief, self-report scale, which was originally developed to measure Generalized Anxiety Disorder, and was validated in primary care settings of the United States [11]. Researchers have also extended the GAD-7 to screen for other anxiety disorders (e.g., Post-Traumatic Stress Disorder, Social Anxiety Disorder, and Panic Disorder) and found that the GAD-7 performed well for most of the anxiety disorders studied [10,12]. With the increasing demand for easy-to-use and effective anxiety scales in various countries, the GAD-7 has been translated into different languages (e.g., Chinese) over the two decades [13,14,15,16]. The GAD-7 has gradually become a widely used measurement tool in populations with different characteristics, especially adult populations, and showed satisfactory reliability and validity [17,18].

Although the GAD-7 has also been used for anxiety screening among adolescents, only a few studies have evaluated its measurement properties in adolescent samples. In a study on adolescents (average age = 16.85 years) in Ghana, the GAD-7 showed adequate internal consistency, a unidimensional structure, and criterion validity [19]. In a recent study using survey data from 111,171 Finnish adolescents aged 14–18 years, the GAD-7 demonstrated good psychometric properties, including good internal consistency, a unidimensional factor structure, and criterion validity [20]. In another recent study on young Americans aged 14–26 years with substance use disorders, the GAD-7 also showed excellent internal consistency and construct validity [21]. However, to the best of our knowledge, there are no psychometric tests of the GAD-7 in Chinese adolescents. The GAD-7 has been translated to Chinese and validated in Chinese outpatients in a general hospital in 2010 [22]. Since then, the GAD-7 has been validated in other Chinese adult samples in hospital settings [15,23,24] and university students [25,26]. Given that adolescents may differently comprehend a questionnaire’s items in the same way as adults do, examining the psychometric properties of the GAD-7 in adolescents is essentially needed.

Additionally, substantial evidence has indicated that female adolescents are more likely to develop anxiety disorders than males [5,8,27]. Prior research has also found that the prevalence of anxiety varies across different periods of adolescence, with more common anxiety symptoms observed in older adolescents [5,27]. However, to date, few studies have tested psychometric properties for the GAD-7 by sex and covered adolescents in early to late adolescence. Previous studies have shown that psychometric properties of the GAD-7 were similar across sex or age groups in Ghanian adolescents [19], Finnish adolescents [20], and Canadian youth [28]. All these studies found that GAD-7 scores were higher in females and older adolescents. Nevertheless, the generalizability of these study results to other countries remains unknown, given that discrepancy in the perception of mental distress such as anxiety across cultures has been observed [29,30]. Therefore, sex- and age-stratified analyses are needed in examining the GAD-7 in Chinese adolescents.

Using a large sample of more than 60,000 Chinese adolescents, this study had the opportunity to examine the internal consistency, factor structure, and criterion validity of the GAD-7 by sex- and age-stratified analyses. As depression symptoms are frequently found to be comorbid with anxiety symptoms [8,31], we adopted depression scores assessed by the Patient Health Questionnaire-9 item (PHQ-9) as a criterion [32] when examining the criterion validity of the GAD-7. Criterion validity refers to the degree to which two measures of constructs theoretically should be related, also known as convergent validity. The PHQ-9 was adopted to measure depression because it is an easy-to-use and widely used tool and has been proved to have satisfactory psychometric properties in the Chinese general population [33], as well as Chinese adolescents [34]. Existing literature has demonstrated that the GAD-7 was highly and positively correlated with the PHQ-9 in adolescents [19,20]. Specifically, we expected that the GAD-7 would present good internal consistency, a unidimensional structure, and a high and positive correlation with the PHQ-9 across sex and age groups. This study would provide evidence for whether the GAD-7 is optimal for measuring anxiety symptoms in Chinese adolescents of different sex and age groups.

## 2. Methods

### 2.1. Procedure and Participants

Data from this study were from a large-scale sampling survey in March 2021 in Shenzhen, one of the most economically developed cities in China. In cooperation with the Educational Science Research Institute of Shenzhen, this study used a multistage sampling design to target students in local primary and middle schools in each district of Shenzhen. All targeted schools are public schools that are under the unified management of the Shenzhen Education Bureau and the guidance of the Ministry of Education of the People’s Republic of China. The inclusion criteria were as follows: students (1) enrolled in Grade 5 or 6 in primary schools, or Grade 1 or 2 in junior high schools, or Grade 1 or 2 in high schools (we did not include students in Grade 3 in junior high schools and high schools because they were about to take the high school or college entrance examination and may not be able to coordinate the time to complete such a large-scale survey); (2) who had the ability to read and understand Chinese well; and (3) who agreed to participate in the study after being fully informed of the details of the study. The exclusion criteria were the student himself/herself, his/her guardians, or teachers disagreed with the student participating in the survey or thought that the student was not suitable to participate in the survey. We imported our questionnaire into the Wenjuanxing platform (a Chinese online survey platform, https://www.wjx.cn/, accessed on 8 December 2021) to facilitate students to fill in and submit the questionnaire. The background and purpose of the survey, as well as informed consent, were explained on the first page of the questionnaire. All participants and their guardians and school principals were informed of the survey. Students who agreed to participate in the study completed the online questionnaire (which took about 20 min) with the class as a unit on school days under the supervision and instruction of a teacher or school staff.

A total of 78,428 questionnaires from 135 schools were collected. After excluding invalid questionnaires (e.g., did not submit the questionnaire within the allotted time, had unidentifiable information, or gave excessive repetitive responses), valid questionnaires from 67,821 adolescents aged 10–17 years were obtained and included in the analysis. More details on participants’ characteristics can be found in Supplementary Materials (Table S1). The Institutional Research Ethics Board at Shenzhen university approved the data collection (Grant number: 2020005).

### 2.2. Measures

Each study participant was required to report his/her basic personal information, including sex (male or female) and age (years). Then, the GAD-7 was prepared to assess participants’ anxiety symptoms over the past 2 weeks [11]. We used the Chinese version translated by He et al. [22]. The consent to introduce the scale to China was obtained from the original authors of the GAD-7. After a series of translations and back translations, three professional researchers and one chief psychiatrist proficient in English formed the final version. The GAD-7 consists of seven items about worry or somatic tension, including (1) Feeling nervous, anxious, or on edge; (2) Becoming easily annoyed or irritable; (3) Feeling afraid as if something awful might happen; (4) Worrying too much about different things; (5) Being so restless that it is hard to sit still; (6) Not being able to stop or control worrying; and (7) Trouble relaxing. The responses of all the items of the GAD-7 are graded on a 4-point Likert scale as 0 (not at all), 1 (several days), 2 (more than half the days), and 3 (nearly every day). Therefore, total scores of the GAD-7 range from 0 to 21, with the higher scores indicating higher severity of anxiety disorders. For the cutoff points of the GAD-7 scores, in general, the scores of 5, 10, and 15 match categorization of none/normal (0–4), mild anxiety (5–9), moderate anxiety (10–14), and severe anxiety (15–21), respectively [11]. The Patient Health Questionnaire 9-item (PHQ-9) was used to assess depression symptoms in study participants [32]. In previous studies on Chinese general and adolescent populations [33,34], the PHQ-9 showed good psychometric properties. In this study, the Cronbach’s α coefficients of the PHQ-9 ranged from 0.89–0.92 across sex- and age-specific groups, showing excellent internal consistency.

### 2.3. Statistical Analysis

Continuous variables were described as means and standard deviations (SD), and categorical variables were described as counts (*n*) with percentages (%). The *t*-test was used to examine the sex age differences of GAD-7 scores. A linear trend between the age and GAD-7 sum score was tested in the analysis of covariance (ANOVA). For reliability, the internal consistency of the GAD-7 was tested using Cronbach’s α coefficients, with values greater than 0.70 that were considered acceptable for research purposes [35]. Sampling adequacy for study participants by sex and age individually was determined with Kaiser–Meyer–Olkin (KMO) testing. To validate that the GAD-7 items were inter-correlated and applicable for factor analysis procedures, Bartlett’s test of sphericity was conducted. Principal component analysis (PCA) was accomplished to explore the factor structure of the GAD-7. Factor loading > 0.6 is considered acceptable [36]. To assess how well the one-factor CFA model fitted the data, five indicators calculated by confirmatory factor analysis (CFA) were comparative fit index (CFI), Tucker–Lewis index (TLI), goodness of fit index (GLI), root mean square error of approximation (RMSEA), and standardized root mean-square residual (SRMR). All indicators range from 0 to 1, with values of CFI, TLI, and TLI closer to 1 signifying better fit and RMSEA and SRMR near 0 indicating a better fit [37]. Specifically, we expected CFI and TLI to be larger than 0.95, GFI to be larger than 0.90, RMSEA to be smaller than 0.08, and SRMR to be smaller than 0.10. Criterion validity was determined by correlating GAD-7 scores with PHQ-9 scores (Spearman rank-order correlations). The significance level for all analyses was set at *p* < 0.05.

## 3. Results

### 3.1. Sample Characteristics

As shown in Table 1, our sample included 67,281 adolescents aged 10–17 years (13.0 ± 1.8), including 51.9% males and 48.1% females. More socio-demographic information about participants is provided in Supplementary Materials (Table S1).

**Figure 1:**
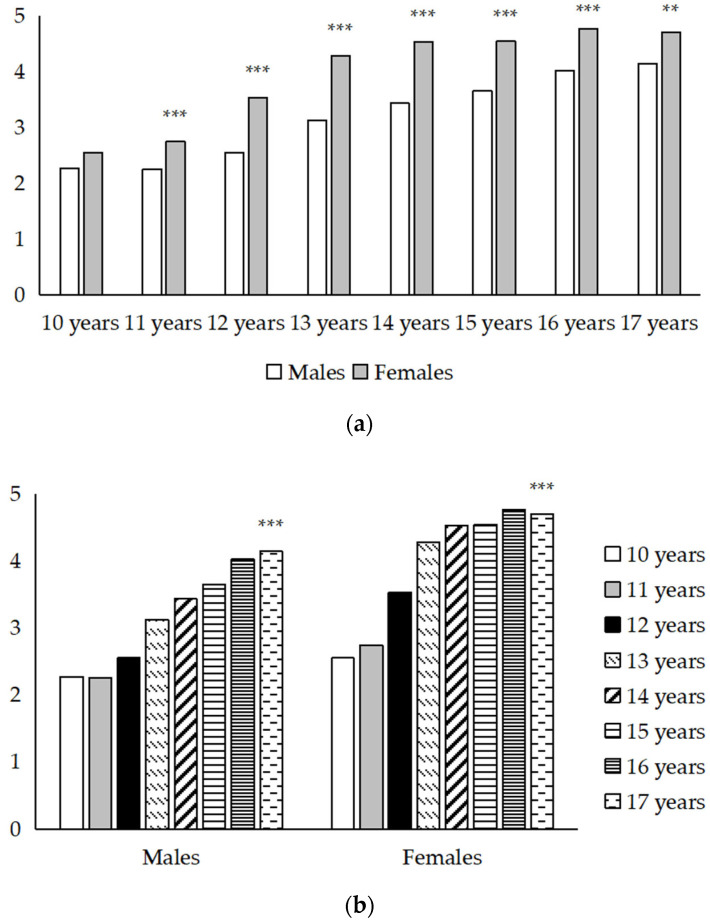
(**a**) Sex differences in total GAD-7 scores by age. (**b**) Age differences in total GAD-7 scores by sex. Values of the Y-axis represent total scores of the GAD-7. ** *p* < 0.01, *** *p* < 0.001.

As shown in Figure 1, independent *t*-tests revealed that except for the 10 years’ age group (*p* = 0.094), females scored significantly higher on the GAD-7 than males in the 11 to 17 years’ age groups (all *p* ≤ 0.001). Additionally, the linear trend test showed that as the age increased, the total GAD-7 scores reported by adolescents increased in both sexes, presenting a significant linear trend (*p* < 0.001). Descriptive characteristics stratified by sex and age for the GAD-7 items and for the whole scale are summarized in Supplementary Materials (Table S2).

### 3.2. Reliability

Table 2 presents corrected item-total correlations ranging from 0.70 to 0.88 (males: 0.71 to 0.88, females: 0.70–0.88). Intercorrelations between the GAD-7 items ranged from 0.55 to 0.85 (males: 0.58 to 0.84, females: 0.55 to 0.85), provided in Supplementary Materials (Tables S3 and S4).

The internal consistency of the GAD-7 was good in all the subgroups (Table 3), with Cronbach’s α coefficients ranging from 0.93 to 0.95 (males: 0.93 to 0.95, females: 0.94 to 0.95).

### 3.3. Factor Structure

The one-factor structure of the GAD-7 was supported by the PCA. Prior to the PCA, KMO results by each subgroup were determined, with all KMO values >0.9, indicating that the sample size for all subgroups was sufficient. The significance (*p* < 0.0001) of Bartlett’s test of sphericity was found across all the subgroups, suggesting that the GAD-7 items were correlated and fitted for factor analysis in all subgroups. Table 4 shows the loadings of seven items on the single factor, with loading values ranging from 0.78 to 0.92 (males: 0.78 to 0.92, females: 0.78 to 0.91).

As Table 5 shows, the unidimensional model fitted the data quite well. Goodness-of-fit results indicated a good to excellent model fit for both males and females in all age groups. Specifically, CFI ranged from 0.96 to 0.99 (males: 0.97 to 0.98, females: 0.96 to 0.99); TLI ranged from 0.95 to 0.98 (males: 0.95 to 0.97, females: 0.96 to 0.98); and GFI ranged from 0.93 to 0.98 (males: 0.93 to 0.97, females: 0.94 to 0.98). RMSEA was somewhat larger than the generally recommended acceptable fit cutoff (males: 0.09 to 0.12, females: 0.07 to 0.13). SRMR was ideal in all subgroups, ranging from 0.02 to 0.04 (males: 0.03 to 0.04, females: 0.02 to 0.04).

### 3.4. Criterion Validity

As Table 6 presents, Spearman rank-order correlations indicated that GAD-7 scores correlated with PHQ-9 scores in all subgroups, with correlation coefficients ranging from 0.70 to 0.82 (males: 0.70 to 0.79, females: 0.76 to 0.82) (all *p* < 0.01).

## 4. Discussion

As mental health problems in adolescents have been a common public health issue [3,38], regular and simple screening tools for specific mental health problems, such as depression and anxiety disorders, are becoming increasingly needed. In this study, we gained convincing evidence that the GAD-7 is a reliable and valid self-reported measure with good psychometric properties in Chinese adolescents aged 10 to 17 years old. To our knowledge, this study is the first one to investigate the psychometric properties of the GAD-7 in Chinese adolescents, which adds evidence that the GAD-7 is a feasible screening tool to identify anxiety symptoms at the population level.

As expected, we apparently found sex and age differences in the GAD-7 scores. Specifically, the GAD-7 scores were higher in females than males, and the GAD-7 scores presented a significant increasing trend as age increased. A study examining the GAD-7 in Finnish adolescents also found that GAD-7 scores were the lowest in the youngest age group (14 years) and the highest in the oldest age group (18 years), although a linear association between the GAD total score and age was not supported in this Finnish study [20]. The disparity of findings between our study and the Finnish study may be because our study covered a larger age range and, thus, was able to detect the linear trend of increased GAD-7 scores with age.

The Cronbach’s α coefficients of the GAD-7 in adolescents by sex and age ranged from 0.93–0.95, indicating excellent internal consistency. This research finding paralleled the early research in which the Cronbach’s alpha coefficients were at good to excellent levels in different populations [20,21,23,26,39]. In our study, Cronbach’s α was at least 0.93 regardless of groups by sex and age; thus, excellent internal reliability of the GAD-7 was verified in Chinese adolescents as well.

The findings of the PCA in this study paralleled previous studies on the GAD-7 based on young adult samples [26,39,40]. Specifically, the results for factor loadings revealed a unidimensional structure with strong loadings (all factor loadings values > 0.6). Furthermore, the results for CFA in sex- and age-specific subgroups were highly similar to those studies that reported good goodness-of-fit indices in the overall sample [40,41]. These research findings alongside prior studies indicate that the GAD-7 has good cultural adaptations in different populations. Of note, to our knowledge, our study is the first to investigate the goodness-of-fit of the GAD-7 in adolescents by sex- and age-specific subgroups, which provide persuasive evidence for the applicability of the GAD-7 in different young populations.

When looking at criterion validity, the GAD-7 and the PHQ-9 should be expectedly related, as anxiety and depression co-occur frequently. In this study, the GAD-7 and PHQ-9 scores presented strong correlations (all correlation coefficients in different subgroups > 0.7). Such a correlation between these two mental health problem measures supports some empirical research exploring the comorbidity between anxiety and depression symptoms (measured by the GAD-7 and PHQ-9). For example, increasing studies found that the symptoms of anxiety (measured by the GAD-7) and depression (measured by the PHQ-9) are intercorrelated and directly interacted [42,43,44]. Moreover, researchers have found the GAD-7 performed well beyond measuring generalized anxiety disorder, but is also a useful measure in the mixed anxiety–depression sample [12]. As criterion validity can also be viewed as an indicator to assess criterion validity, we can deem that the criterion validity of the GAD-7 is acceptable in Chinese adolescents.

This study has some strengths. To the authors’ knowledge, the current study is the first to investigate the psychometrics of the GAD-7 in an exclusively Chinese adolescent sample with a large sample size (*n* > 60,000). Further, we examined the reliability and validity of the GAD-7 in adolescents in sex- and age-specific subgroups, which provided robust evidence on the psychometric performance of applying GAD-7 in adolescents. This strength can help demonstrate the GAD-7 is a feasible instrument across different sexes and ages in young people.

However, a key limitation of the current study was that our study lacked diagnosis-specific criterion measures to assess the criterion validity of the GAD-7 since diagnostic interviews were not conducted in this study, and we only assessed the criterion validity by combining the PHQ-9. We noted that one study published in 2017 found that the specificity and sensitivity for detecting clinically significant anxiety symptoms were acceptable in adolescents with generalized anxiety disorder in Cincinnati [45]. However, the study used a small sample size and all patients were evaluated at a single site, thus potentially limiting generalizability. Although clinical utility of the GAD-7 has been verified in adults [12,18,23], more validation studies conducted in clinical adolescents are needed. Additionally, due to the lack of a diagnostic criterion, we failed to investigate the suitable cutoff of the GAD-7 in our sex- and age-specific groups. Therefore, further studies to include diagnosis-specific criterion measures and to determine the optimal cutoff point of the GAD-7 in adolescents are still warranted. Additionally, our study was based on the study samples from Shenzhen city, a southern city in mainland China. For this line, our research findings may not be generalizable to other adolescents from different countries or cultures.

## 5. Conclusions

The GAD-7 has been recognized with acceptable properties for screening generalized anxiety disorders in adults and adolescents in some countries (mainly Western countries). It is necessary to test the psychometric properties of the GAD-7 in more populations, such as adolescents in Asian countries (as with the current study). The present study offers evidence concerning the psychometric characteristics of the GAD-7 in Chinese adolescents. We preliminarily found that the GAD-7 is a reliable and valid instrument tool to assess anxiety in Chinese adolescents. Although the results from this study echo earlier studies of other populations, more empirical evidence is needed to assess the diagnostic validity of the instrument for identifying clinical adolescents. This would call for more studies with improved study design (e.g., including diagnostic interview) to determine the reliability and validity as well as the clinical utility of the GAD-7 in adolescent populations.

## Figures and Tables

**Table 1:** Sample size by subgroups.

Age	Total		Males		Females	
	*n*	%	*n*	%	*n*	%
10 years	2430	3.6	1224	3.5	1206	3.7
11 years	10,933	16.2	5743	16.5	5190	16.0
12 years	16,244	24.1	8745	25.1	7499	23.2
13 years	15,331	22.8	7943	22.8	7388	22.8
14 years	8871	13.2	4769	13.7	4102	12.7
15 years	4076	6.1	1962	5.6	2114	6.5
16 years	6379	9.5	3014	8.6	3365	10.4
17 years	3017	4.5	1509	4.3	1508	4.7

**Table 2:** Corrected item-total correlations by subgroups.

Age	Males							Females						
	Item 1	Item 2	Item 3	Item 4	Item 5	Item 6	Item 7	Item 1	Item 2	Item 3	Item 4	Item 5	Item 6	Item 7
10 years	0.80	0.79	0.81	0.78	0.74	0.74	0.72	0.81	0.87	0.85	0.84	0.75	0.80	0.78
11 years	0.79	0.81	0.81	0.79	0.76	0.76	0.71	0.81	0.83	0.84	0.83	0.76	0.78	0.76
12 years	0.81	0.82	0.83	0.81	0.77	0.78	0.73	0.82	0.86	0.85	0.84	0.76	0.79	0.77
13 years	0.80	0.82	0.83	0.80	0.78	0.77	0.75	0.83	0.86	0.85	0.83	0.76	0.80	0.76
14 years	0.81	0.84	0.84	0.83	0.79	0.78	0.77	0.83	0.87	0.86	0.85	0.76	0.79	0.77
15 years	0.79	0.84	0.84	0.82	0.77	0.77	0.76	0.84	0.88	0.85	0.84	0.75	0.80	0.75
16 years	0.84	0.86	0.85	0.84	0.80	0.81	0.76	0.83	0.87	0.85	0.84	0.74	0.80	0.74
17 years	0.84	0.88	0.85	0.83	0.81	0.80	0.75	0.80	0.87	0.85	0.84	0.72	0.81	0.70

**Table 3:** Internal consistency by subgroups (indicated by Cronbach’s α coefficients).

Age	Males	Females
10 years	0.93	0.94
11 years	0.93	0.94
12 years	0.94	0.94
13 years	0.94	0.94
14 years	0.94	0.95
15 years	0.94	0.94
16 years	0.95	0.94
17 years	0.95	0.94

**Table 4:** Principal components analysis factor loadings of items by subgroups.

Age	Males							Females						
	Item 1	Item 2	Item 3	Item 4	Item 5	Item 6	Item 7	Item 1	Item 2	Item 3	Item 4	Item 5	Item 6	Item 7
10 years	0.86	0.86	0.87	0.85	0.81	0.81	0.79	0.86	0.91	0.89	0.88	0.82	0.86	0.84
11 years	0.85	0.87	0.87	0.85	0.82	0.83	0.78	0.87	0.88	0.89	0.88	0.82	0.84	0.82
12 years	0.86	0.88	0.88	0.87	0.83	0.84	0.80	0.87	0.90	0.89	0.89	0.83	0.85	0.83
13 years	0.86	0.87	0.88	0.86	0.84	0.83	0.81	0.88	0.90	0.90	0.88	0.82	0.85	0.83
14 years	0.87	0.89	0.89	0.88	0.85	0.84	0.83	0.88	0.91	0.90	0.89	0.82	0.85	0.83
15 years	0.85	0.89	0.89	0.87	0.83	0.83	0.82	0.89	0.91	0.89	0.89	0.81	0.85	0.82
16 years	0.88	0.9	0.89	0.89	0.86	0.86	0.82	0.88	0.91	0.89	0.89	0.81	0.85	0.81
17 years	0.89	0.92	0.89	0.88	0.86	0.86	0.81	0.86	0.91	0.89	0.89	0.79	0.86	0.78

**Table 5:** Goodness-of-fit indices by subgroups.

Age	Males					Females				
	CFI	TLI	GFI	RMSEA	SRMR	CFI	TLI	GFI	RMSEA	SRMR
10 years	0.97	0.96	0.95	0.10	0.03	0.98	0.98	0.97	0.09	0.02
11 years	0.97	0.96	0.96	0.10	0.03	0.98	0.97	0.96	0.10	0.03
12 years	0.98	0.97	0.96	0.09	0.03	0.99	0.98	0.98	0.07	0.02
13 years	0.98	0.97	0.97	0.09	0.03	0.98	0.98	0.97	0.08	0.02
14 years	0.98	0.97	0.96	0.10	0.03	0.98	0.97	0.96	0.10	0.03
15 years	0.97	0.95	0.94	0.11	0.03	0.98	0.96	0.95	0.10	0.03
16 years	0.97	0.95	0.93	0.12	0.03	0.97	0.96	0.95	0.11	0.03
17 years	0.97	0.95	0.94	0.12	0.04	0.96	0.96	0.94	0.13	0.04

Note. CFI = comparative fit index; TLI = Tucker–Lewis index; GFI = goodness of fit index; RMSEA = root mean square error of approximation; SRMR = standardized root mean-square residual.

**Table 6:** Correlations between total GAD-7 scores and PHQ-9 scores by subgroups.

Sex	10 Years	11 Years	12 Years	13 Years	14 Years	15 Years	16 Years	17 Years
Males	0.70 **	0.74 **	0.75 **	0.76 **	0.77 **	0.77 **	0.79 **	0.78 **
Females	0.76 **	0.77 **	0.81 **	0.82 **	0.82 **	0.80 **	0.77 **	0.77 **

Note. ** *p* < 0.01.

## Data Availability

The datasets analyzed in the current study are available from the corresponding author on reasonable request.

## Supplementary Materials

The following are available online at https://www.mdpi.com/article/10.3390/healthcare9121709/s1, Table S1. Participants’ socio-demographic information; Table S2. Descriptive characteristics of item scores and total scores by subgroups; Table S3. Correlation between items in male adolescents by age groups; Table S4. Correlation between items in female adolescents by age groups.
