# Supplementary material for: Psychometric Properties of the Generalized Anxiety Disorder Scale-7 Item (GAD-7) in a Large Sample of Chinese Adolescents
Source: Healthcare (Basel). 2021 Dec 9;9(12):1709. doi: 10.3390/healthcare9121709 (PMC8701121; doi:10.3390/healthcare9121709)
Supplement: Supplementary file 1 [file healthcare-09-01709-s001.zip › healthcare-1469454-supplementary.pdf]

healthcare

MDPI

Table S1. Participants' socio-demographic information.

Variables		*N* (%)
Gender	Boys	34,909 (51.9)
	Girls	32,372 (48.1)
Grade	Primary school	27,954 (41.5)
	Junior middle school	27,124 (40.3)
	High school	12,203 (18.1)
Puberty status	Non-pubertal	33,288 (49.5)
	Pubertal	33,993 (50.5)
Weight status	Normal	45,817 (68.1)
	Overweight	9051 (13.5)
	Obese	12,413 (18.4)
Siblings	Only child	17,354 (25.8)
	Non-only child	49,927 (74.2)
Family structure	Full	62,836 (93.4)
	Other	4445 (6.6)
Paternal education	Junior middle school or below	14,619 (21.7)
	High school or equivalent	18,159 (27.0)
	Bachelor or equivalent	26,030 (38.7)
	Master or above	2796 (4.2)
	Unclear	5677 (8.4)
Maternal education	Junior middle school or below	17,617 (26.2)
	High school or equivalent	18,706 (27.8)
	Bachelor or equivalent	23,922 (35.6)
	Master or above	1635 (2.4)
	Unclear	5401 (8.0)
Nationality	Han	65,027 (96.6)
	minority	2254 (3.4)

Attending schools in Shenzhen since childhood	Yes	61,969 (92.1)
	No	5312 (7.9)
**Variables**		**Mean (SD)**
Age (years)		13.0 (1.8)
Subjective family socioeconomic status		5.0 (1.7)

Note: SD: standard deviation.

Table S2. Descriptive characteristics of item scores and total scores by subgroups.

Item	Age	Males		Females		Age	Males		Females	
		Mean	SD	Mean	SD		Mean	SD	Mean	SD
Item 1	10 years	0.33	0.64	0.37	0.66	14 years	0.52	0.73	0.70	0.82
Item 2		0.28	0.63	0.32	0.67		0.44	0.74	0.64	0.85
Item 3		0.33	0.68	0.36	0.71		0.54	0.80	0.74	0.90
Item 4		0.34	0.70	0.38	0.74		0.53	0.81	0.69	0.89
Item 5		0.27	0.62	0.26	0.60		0.39	0.70	0.43	0.73
Item 6		0.37	0.69	0.43	0.76		0.53	0.80	0.74	0.91
Item 7		0.36	0.74	0.43	0.78		0.48	0.80	0.60	0.86
Total scores		2.27	3.92	2.55	4.25		3.43	4.63	4.53	5.16
Item 1	11 years	0.32	0.62	0.39	0.68	15 years	0.57	0.73	0.73	0.79
Item 2		0.27	0.61	0.36	0.69		0.50	0.75	0.68	0.83
Item 3		0.33	0.67	0.42	0.73		0.6	0.78	0.78	0.86
Item 4		0.34	0.70	0.41	0.75		0.58	0.82	0.70	0.84
Item 5		0.27	0.62	0.28	0.61		0.41	0.68	0.43	0.68
Item 6		0.36	0.69	0.45	0.77		0.54	0.76	0.70	0.84
Item 7		0.35	0.73	0.44	0.78		0.45	0.73	0.53	0.78
Total scores		2.25	3.88	2.74	4.29		3.65	4.50	4.54	4.87
Item 1	12 years	0.39	0.68	0.52	0.75	16 years	0.64	0.76	0.78	0.8
Item 2		0.32	0.65	0.47	0.77		0.57	0.76	0.72	0.83
Item 3		0.38	0.71	0.55	0.81		0.67	0.80	0.81	0.85
Item 4		0.38	0.73	0.53	0.83		0.64	0.80	0.73	0.84
Item 5		0.29	0.64	0.35	0.67		0.45	0.71	0.47	0.71

Item 6		0.40	0.73	0.59	0.84		0.57	0.78	0.74	0.83
Item 7		0.39	0.75	0.52	0.83		0.47	0.75	0.52	0.78
Total scores		2.55	4.15	3.53	4.75		4.02	4.67	4.76	4.87
Item 1	13 years	0.48	0.71	0.65	0.8	17 years	0.67	0.77	0.77	0.76
Item 2		0.39	0.70	0.60	0.84		0.6	0.78	0.70	0.81
Item 3		0.48	0.76	0.70	0.88		0.69	0.82	0.81	0.83
Item 4		0.47	0.78	0.65	0.88		0.65	0.84	0.73	0.82
Item 5		0.36	0.68	0.41	0.7		0.47	0.73	0.45	0.66
Item 6		0.50	0.78	0.70	0.88		0.58	0.78	0.73	0.80
Item 7		0.44	0.78	0.57	0.85		0.49	0.74	0.51	0.74
Total scores		3.12	4.42	4.28	5.06		4.14	4.76	4.70	4.63

Note: SD = standard deviations.

Table S3. Correlation of between items in male adolescents by age groups.

Age	Item	Item 1	Item 2	Item 3	Item 4	Item 5	Item 6	Item 7
10 years	Item 1	1						
	Item 2	0.74	1					
	Item 3	0.74	0.76	1				
	Item 4	0.68	0.67	0.69	1			
	Item 5	0.62	0.6	0.62	0.64	1		
	Item 6	0.65	0.6	0.6	0.65	0.67	1	
	Item 7	0.59	0.61	0.64	0.6	0.6	0.59	1
11 years	Item 1	1						
	Item 2	0.76	1					
	Item 3	0.73	0.75	1				
	Item 4	0.67	0.71	0.7	1			
	Item 5	0.61	0.63	0.63	0.65	1		
	Item 6	0.64	0.65	0.64	0.66	0.69	1	
	Item 7	0.58	0.6	0.63	0.6	0.62	0.59	1
12 years	Item 1	1						
	Item 2	0.77	1					

	Item 3	0.75	0.77	1				
	Item 4	0.7	0.73	0.73	1			
	Item 5	0.64	0.66	0.66	0.68	1		
	Item 6	0.66	0.66	0.67	0.69	0.71	1	
	Item 7	0.62	0.63	0.67	0.63	0.62	0.63	1
	Item 1	1						
	Item 2	0.75	1					
	Item 3	0.74	0.78	1				
13 years	Item 4	0.7	0.71	0.74	1			
	Item 5	0.65	0.67	0.66	0.66	1		
	Item 6	0.66	0.65	0.67	0.66	0.69	1	
	Item 7	0.62	0.64	0.66	0.64	0.65	0.64	1
	Item 1	1						
	Item 2	0.78	1					
	Item 3	0.75	0.79	1				
14 years	Item 4	0.72	0.75	0.77	1			
	Item 5	0.68	0.68	0.67	0.71	1		
	Item 6	0.67	0.68	0.67	0.7	0.71	1	
	Item 7	0.64	0.68	0.69	0.68	0.69	0.65	1
	Item 1	1						
	Item 2	0.75	1					
	Item 3	0.73	0.82	1				
15 years	Item 4	0.71	0.75	0.77	1			
	Item 5	0.62	0.66	0.65	0.68	1		
	Item 6	0.64	0.67	0.66	0.67	0.69	1	
	Item 7	0.62	0.66	0.66	0.64	0.67	0.67	1
	Item 1	1						
16 years	Item 2	0.8	1					
	Item 3	0.78	0.83	1				
	Item 4	0.76	0.77	0.78	1			

	Item 5	0.68	0.71	0.68	0.71	1		
	Item 6	0.71	0.71	0.7	0.73	0.76	1	
	Item 7	0.65	0.67	0.67	0.66	0.7	0.67	1
17 years	Item 1	1						
	Item 2	0.84	1					
	Item 3	0.79	0.82	1				
	Item 4	0.76	0.78	0.79	1			
	Item 5	0.68	0.73	0.68	0.7	1		
	Item 6	0.68	0.73	0.7	0.69	0.77	1	
	Item 7	0.65	0.68	0.66	0.64	0.69	0.67	1

Table S4. Correlation of between items in female adolescents by age groups.

Age	Item	Item 1	Item 2	Item 3	Item 4	Item 5	Item 6	Item 7
10 years	Item 1	1.00						
	Item 2	0.74	1.00					
	Item 3	0.74	0.84	1.00				
	Item 4	0.72	0.77	0.74	1.00			
	Item 5	0.65	0.69	0.66	0.68	1.00		
	Item 6	0.69	0.73	0.69	0.71	0.69	1.00	
	Item 7	0.67	0.72	0.72	0.71	0.60	0.68	1.00
11 years	Item 1	1.00						
	Item 2	0.77	1.00					
	Item 3	0.74	0.79	1.00				
	Item 4	0.72	0.75	0.77	1.00			
	Item 5	0.65	0.66	0.65	0.67	1.00		
	Item 6	0.67	0.67	0.67	0.68	0.69	1.00	
	Item 7	0.64	0.64	0.68	0.66	0.63	0.67	1.00
12 years	Item 1	1.00						
	Item 2	0.78	1.00					
	Item 3	0.75	0.81	1.00				
	Item 4	0.73	0.77	0.77	1.00			

	Item 5	0.66	0.68	0.67	0.69	1.00		
	Item 6	0.68	0.70	0.71	0.71	0.67	1.00	
	Item 7	0.65	0.69	0.69	0.69	0.65	0.68	1.00
13 years	Item 1	1.00						
	Item 2	0.80	1.00					
	Item 3	0.76	0.82	1.00				
	Item 4	0.73	0.77	0.77	1.00			
	Item 5	0.66	0.68	0.65	0.68	1.00		
	Item 6	0.70	0.71	0.71	0.71	0.68	1.00	
	Item 7	0.65	0.68	0.69	0.67	0.65	0.67	1.00
14years	Item 1	1.00						
	Item 2	0.80	1.00					
	Item 3	0.77	0.84	1.00				
	Item 4	0.75	0.78	0.79	1.00			
	Item 5	0.66	0.68	0.65	0.69	1.00		
	Item 6	0.69	0.71	0.70	0.72	0.68	1.00	
	Item 7	0.67	0.69	0.69	0.68	0.66	0.66	1.00
15 years	Item 1	1.00						
	Item 2	0.81	1.00					
	Item 3	0.77	0.84	1.00				
	Item 4	0.75	0.79	0.79	1.00			
	Item 5	0.66	0.67	0.63	0.66	1.00		
	Item 6	0.70	0.71	0.71	0.70	0.70	1.00	
	Item 7	0.66	0.68	0.67	0.66	0.64	0.65	1.00
16 years	Item 1	1.00						
	Item 2	0.81	1.00					
	Item 3	0.77	0.83	1.00				
	Item 4	0.76	0.79	0.78	1.00			
	Item 5	0.63	0.66	0.62	0.66	1.00		
	Item 6	0.69	0.72	0.71	0.70	0.69	1.00	

	Item 7	0.63	0.67	0.66	0.64	0.65	0.65	1.00
17 years	Item 1	1.00						
	Item 2	0.78	1.00					
	Item 3	0.74	0.85	1.00				
	Item 4	0.74	0.79	0.79	1.00			
	Item 5	0.60	0.62	0.59	0.64	1.00		
	Item 6	0.70	0.73	0.72	0.71	0.64	1.00	
	Item 7	0.55	0.62	0.63	0.61	0.65	0.63	1.00
